# Effects of a Two-Step Cognitive and Relaxation Training Program in Care Home Residents with Mild Cognitive Impairment

**DOI:** 10.3390/ijerph19148316

**Published:** 2022-07-07

**Authors:** Kristina Stuerz, Sabine Hartmann, Bernhard Holzner, Carina S. Bichler, Martin Niedermeier, Martin Kopp, Verena Guenther

**Affiliations:** 1 Department of Psychiatry, Psychotherapy, Psychosomatics and Medical Psychology, University Hospital for Psychiatry I, Tyrol Clinics, Medical University of Innsbruck, 6020 Innsbruck, Austria; kristina.stuerz@tirol-kliniken.at (K.S.); hartmann_s@gmx.at (S.H.); bernhard.holzner@tirol-kliniken.at (B.H.); verena.guenther@mailbox.org (V.G.); 2 Department of Sport Science, University of Innsbruck, 6020 Innsbruck, Austria; martin.niedermeier@uibk.ac.at (M.N.); martin.kopp@uibk.ac.at (M.K.)

**Keywords:** mild cognitive impairment, relaxation training, cognitive training, cognitive performance, well-being

## Abstract

The aim of the present study was to assess the impact of a relaxation training program (RT), a cognitive training program (CT), and the combination of both on changes in cognitive status, emotional status, and experience of pain in older adults with mild cognitive impairment (MCI). Fifty care home residents underwent either RT (26 participants) or CT (24 participants) in the first training period, followed by the combined relaxation and cognitive training program in the second. Psychological tests on cognitive performance, mood disturbance/well-being, depression, and experience of pain were implemented at three time points of measurement, before (t1), after (t2) the first training period, and after the second training period (t3). Both RT and CT with the subsequent combined training program in the second training period, respectively, increased cognitive performance and well-being, and reduced mood disturbance, depression, and the experience of pain. The study showed the non-inferiority of RT in respect of cognitive and emotional status in care home residents with MCI compared to the more frequently implemented CT. Both training programs are high in acceptability and positive outcomes on cognitive, emotional, and pain status support the use of a combination of RT and CT.

## 1. Introduction

Mild cognitive impairment (MCI) obtained increasing attention in clinical practice and research settings [1]. The clinical prodrome MCI manifests as an intermediate state of cognitive function between the aging related changes and those fulfilling the criteria for dementia and Alzheimer’s disease (AD) [1]. Approximately ten to twelve percent of the population which is affected by MCI develops AD within one year, compared to one to two percent of unaffected older adults [2]. In the longer term, 80% of the population affected with MCI is going to develop AD within six years [1,3]. Furthermore, it has been shown that depression can reinforce the transition from MCI to AD [4]. Early treatment of MCI can considerably delay the development of AD and therefore support cognitive and emotional health. In particular, persons who can no longer care for themselves and live in care homes have a high incidence of MCI and preventive interventions are of high interest [2].

There are several treatment options to deal with MCI. Treatment focus has shifted to cognitive stimulation, exercise training programs and diet, since no potent evidence has been found for pharmacological treatment options [5,6]. A meta-analysis by Li et al. demonstrated that cognitive interventions are potentially effective methods to enhance cognitive and functional abilities in people suffering from MCI [7]. Hall et al. reported improvement as well as protection of cognitive functioning due to the engagement in meaningful mental stimulation and intellectual activity [8]. Research on relaxation training (RT) also indicated some evidence regarding increased positive affect and interpersonal relationship in older adults [9]. Considering stress, the usage of relaxation techniques for older adults and cognitively impaired people is highly recommended [10,11], particularly if practice is easy to follow, effective, and quickly mastered [12]. Stress is known to affect both physical and psychological conditions, such as performance, health, depressive symptoms, and worrying [13,14]. Data of a large number of older adults revealed an association of perceived stress and poorer memory, processing speed and executive function [15]. The experience of psychological stress was shown to be related to lower cognitive performance in older adults [16]. Above, data obtained from a vast number of studies in animals and humans suggest that chronic or repeated exposure to stress has enduring effects on the physical brain [13]. Especially during aging, human brains are highly vulnerable to the effects caused by stress hormones [13]. Based on these findings, it can be derived that interventions that potentially induce perceived stress and pressure to perform may lead to cognitive decline, even though they were initially designed to enhance cognitive performance [17,18].

Nevertheless, RT has not yet been considered as a treatment option for MCI. Previous research mainly focused on cognitive health parameters, although emotional health was recognized as an important factor in the treatment of MCI [19]. It is recommended to promote the development of coping strategies and motivation in order to increase cognitive function in older adults [15]. It is suggested that randomized, single-blind, active placebo-controlled studies are required to further assess treatment efficacy [19].

The goals of the study were to assess the impact of and analyze the differences between a relaxation training program according to Jacobson (RT) [20] and a cognitive training program (CT) on cognitive and emotional status, experience of pain, and acceptability in care home residents with MCI in the first training period. In a second training period, a combined training program including relaxation and cognitive training was performed and analyzed in respect of impacts on outcome criteria. Previous research in the field of clinical gerontology has shown that stress impairs mental and physiological health. Consequently, best training effects are expected when participants do not experience stress and rather feel relaxed when participating in a training program to enhance cognitive performance. Therefore, we hypothesize best training effects in a combined training program with both relaxation and cognitive training elements.

## 2. Materials and Methods

### 2.1. Design and Procedure

**Figure 1:**
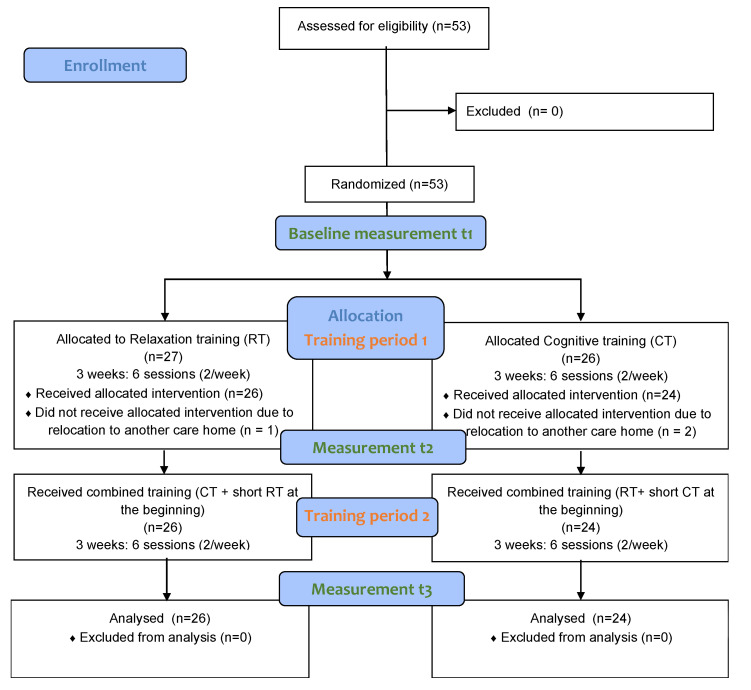
Participant workflow.

The intervention study was designed as a randomized controlled trial to compare the efficacy and acceptability of RT and CT, as well as a combination of both, in participants with MCI. The study took part in three similar structured care homes in Austria. Participants were grouped into clusters of five to eight residents. The clusters were randomized through block randomization (block size: 2) to either in RT or in CT. Both groups received a three-week training program of either relaxation or cognitive training, followed by a second three-week combined training program of both. Every week of the overall six training weeks included two group sessions with a duration of 30 to 35 min each. The total intervention period for each participant comprised six weeks. All participants passed through a test procedure before the training program (t1), after the training program (t2) and after the combined training program (t3) resulting in a 2-factorial group-by-time design. A detailed description of the workflow is presented in Figure 1.

### 2.2. Participants

Fifty-three participants were recruited through requests in care homes, offering a training program for residents with MCI. Nursing personnel selected residents pre-diagnosed by a clinician with MCI, or meeting the criteria of MCI assessed by a MMSE-score ranging from 21–26 points, that could be included in the study due to sufficient visual and auditory ability and cognitive, physiological and psychological capability, based on a clinical valuation. In ambiguous cases regarding study inclusion, an assessment was made by a psychological specialist. Suitable participants, as well as their close dependents, were informed and agreed to participation. Eligible participants provided written informed consent in accordance with the principles of the Declaration of Helsinki, before participating. As the program was an integrated treatment component for participating care home residents, approval by the review board was not required. The training and assessments for both groups were conducted by psychological professionals in a familiar environment of the care home. 

### 2.3. Intervention

#### 2.3.1. Relaxation Training (RT)

Progressive muscle relaxation (PMR) is a relaxation technique by Jacobson [20] to bring calm and rest to the nervous system to induce reciprocity between central nervous and mental mechanisms and further reduce stress and muscular tension [21].

It is an effective technique for managing psychiatric and behavioral disturbance as well as activities of daily living in patients with AD of mild to moderate dementia [22]. As again shown lately, PMR training improves both behavioral and psychological symptoms of dementia, and supports daily living activities in home residents with dementia [23].

PMR-tasks were group-instructed and centered on participants’ individual abilities and needs, accompanied by relaxation music. Listening to relaxation music has previously been shown to improved cognitive functions in healthy older adults [24] and decreased depressive symptoms as well as mental confusion in apoplex patients [25]. PMR combined with listening to relaxation music increased sleep quality compared to PMR only [26]. The easily applicable and empirically evident modification of PMR training by Bernstein et al. was applied [27]. 

#### 2.3.2. Cognitive Training (CT)

A number of cognitive tasks with different difficulty levels were provided as paper and pencil-based training. Cognitive training tasks were instructed by a clinical psychologist and included training manuals for older adults focusing on creativity, perception, concentration, alertness, information processing, short- and long-term memory, fluency of speaking, memory retention, problem solving, and logic [28,29,30]. Moreover, riddle and mental exercise tasks [30] and printed excerpts of the computer-based training program COGPACK were provided. In order to prevent participants from over- or underload, fitting training material and tasks were presented on an individual basis. 

#### 2.3.3. Combined Training 

In the combined training period that followed the first training period, participants began with the training program mastered before (relaxation training or cognitive training) and then continued with the respective other training program. Therefore, RT received a shortened form of PMR followed by cognitive training tasks. CT received cognitive training tasks, followed by a shortened form of PMR. 

### 2.4. Measurements

#### 2.4.1. Sociodemographic Data

Information about participants’ age, sex, education, family status, medical status and level of care was provided from the documentation in the care homes.

#### 2.4.2. Assessment of Effectiveness on Cognitive and Emotional Status

The test procedure was assessed in a single setting before (t1) and after (t2) the training program as well as after the combined training program (t3). The test procedure included screenings of objectively measured cognitive function and subjectively rated depressive mood, well-being and pain. The average session time was 30 to 45 min for each time-point of the test procedure measurement.

(a) Mini-Mental State Examination (MMSE)

The revised Version MMSE-2 [31] is a widely-used screening measurement to assess cognitive deficits in older adults. The MMSE-2 supports the assessment of dementia stages and course of disease in follow-up monitoring. The applied standard version contains thirty items considering timing and spatial orientation, alertness, memory performance, language, and apraxia. The sum score of each correctly answered item (one point) is used to estimate the stage of dementia. A score of 30–27 points is considered as normal, whereas fewer points indicate mild (26–21 points), moderate (20–10 points), or severe (9–0 points) dementia.

(b) Age-Concentration-Test (ACT)

The German versioned Age-Concentration-Test is a psychometric instrument to assess concentration and vigilance in older and demented people [32]. It is language-independent and acquires visual faculty of discrimination and reports required time, right task solving, number of mistakes, percentage of mistakes and overall score. Quality criteria have been verified by Gatterer et al. [32].

(c) Clinical Self-Rating Scale (CSRS-AMS)

The Clinical Self-Rating Scale, developed by the Munich psychiatric information system, includes a subscale to assess the mental state and the extent of subjectively rated mood disturbance [33]. This subscale is called Adjective Mood Scale (AMS) and includes 28 items for the assessment of fluctuations in well-being (or rather its reduction) or mood during psychological and drug treatment, as well as after the accomplishment of training programs [33]. Quality criteria and normative values for German speaking population have been provided [34].

(d) Geriatric Depression Scale-Short Form (GDS-SF)

The short form of the Geriatric Depression Scale (GDS-SF) involves 15 self-reported questions and has found to be an adequate alternative for the original 30-question scale. The GDS-SF assesses (beginning) depressive symptoms in older adults at an early stage [35]. Questions were answered by ‘yes’ or ‘no’ and summed up to a score, where 0–4 assesses no depressive symptoms, 5-10 light to moderate depressive symptoms, and 11–15 severe depressive symptoms. Reliability and validity of the GDS-SF were reported to be sufficient [35].

(e) Visual Analog Scale (VAS) for the Measurement of Experience of Pain

In terms of measuring the subjective experience of pain, the usage of a visual analog scale has been proved successfully and was applied in the study [36]. Information of validity for measuring experience of pain by a VAS was provided by Schomacher [36].

Participants were asked to mark the experienced degree of pain within the last two weeks on a ten-centimeter line, ranging from ‘not at all (0 cm)’ to ‘extremely (10 cm)’. 

#### 2.4.3. Assessment of Acceptability

Participants self-rated the acceptability of their group participation in general, including communication between psychologist and participants, quality and topics of training, group atmosphere, and sense of purpose. The rating is according to the conventional school grade system ranging from ‘1—very good’ to ‘5—insufficient’. Moreover, acceptability of institute leader and care working professionals was assessed using same school grade system ratings.

### 2.5. Statistical Analysis

All statistical analyses were performed using SPSS version 24.0 (IBM, New York, NY, USA). Normal distribution was assessed by Kolmogorov–Smirnov test. Non-normally distributed scale values were logarithmized prior to the application of parametric analyses. For each of the separate outcomes, a 2 × 3 mixed ANOVA was applied to analyze effects of the between-subject factor group (RT, CT), the within-subject factor time (t1, t2, and t3), and group-by-time interactions. A significant group-by-time interaction was interpreted as a different change in the outcomes between groups. In case of a significant interaction, three separate change scores were calculated (change between t1 and t2, t1 and t3, and t2 and t3) and these change scores were compared between groups using *t*-tests for independent samples and Bonferroni correction for multiple comparisons. Data are presented as mean and standard deviation. Partial η^2^ was used as an effect size for the results of the ANOVAs, Cohen’s *d* was calculated for the pairwise comparisons. *p*-Values < 0.05 were regarded as significant (two-way) for the ANOVAs and <0.017 for the Bonferroni corrected post-hoc tests. An effect size for partial η^2^ < 0.06 and Cohen’s *d* < 0.2 was regarded as small, partial η^2^ between 0.06 and 0.14 and Cohen’s *d* between 0.2 and 0.5 as medium, and partial η^2^ > 0.14 and Cohen’s *d* < 0.8 as large.

## 3. Results

### 3.1. Sociodemographic and Clinical Data

Fifty participants (age: M = 87.1 ± 6.3, minimum age 66, maximum age 97, 49 female, 1 male) completed the study in either RT (n = 26) or CT (n = 24), followed by the combined training (see Figure 1). Sociodemographic and clinical data of the participants separated by group is shown in Table 1. The six-week training programs were implemented consecutively, resulting in an overall study duration of 14 months. 

### 3.2. Efficacy of Training Programs

#### 3.2.1. Cognitive Status

Among the dementia screening measured with the MMSE, participants of both groups showed mild impairment in cognitive ability (RT: M = 24.8 ± 3.5; CT: M = 26.3 ± 2.7). A statistically significant group*time interaction effect was found for the MMSE subscale ‘attention and calculation’. Cognitive performance in ‘attention and calculation’ improved significantly more after both training periods in the RT, compared to the CT (*p* = 0.038, partial η^2^ = 0.07). Post-hoc analyses did not reveal statistically significant group changes over time after Bonferroni correction *p* > 0.032, *d* < 0.62. Statistically significant time effects were found for the ‘MMSE total score’, ‘MMSE orientation’, ‘MMSE attention and calculation’, and ‘MMSE recall’, indicating a significant improvement in cognitive performance for RT and CT over the total training period. No statistically significant group, time, or interaction effects were found for the subscales ‘MMSE registration’ and ‘MMSE language’.

For cognitive performance on concentration and vigilance (ACT), a statistically significant group*time interaction effect was found for ‘ACT work pace’ (*p* = 0.026, η^2^ = 0.08). Post-hoc analyses revealed statistically significant improvements in RT after completing the first training period (*p* = 0.010, *d* = 0.75), whereas CT revealed statistically significant improvements after practicing the combined training (*p* = 0.008, *d* = − 0.79). Significant time effects were found for the subscales ‘ACT work pace’ and ‘ACT percentage of mistakes’, indicating improvement over the time of both training periods. No statistically significant group, time, or interaction effects were found for the ‘ACT overall score’ and the subscale ‘ACT correct answer’ (see Table 2, Cognitive Status).

#### 3.2.2. Emotional Status

All participants were located in an unremarkable range of depressive symptoms at baseline (t1) (MW = 3.12, ± 2.97 for RT; MW = 2.83, ± 2.12 for CT). No statistically significant group*time interaction was found for mood disturbance/well-being (CSRS-AMS) or depressive symptoms (GDS-SF). Both groups yielded a significant reduction with large effect size in mood disturbance (*p* < 0.001, partial η^2^ = 0.30) and depressive symptoms (*p* < 0.001, partial η^2^ = 0.28) after completing both training periods. Results can be seen in Table 2 (Emotional Status).

#### 3.2.3. Experience of Pain

Participants in the RT/CT were experiencing 2.8/3.6 on a 0 (minimal pain) to 10 (maximal pain) scale at baseline. No statistically significant group*time interaction was found (*p* = 0.681). A statistically significant time effect was found (*p* = 0.019, η^2^ = 0.08), indicating decreased experience of pain over time for both groups. 

### 3.3. Acceptability

Based on an after-treatment conversation, 78% of participants rated the acceptability of their group participation as ‘very good’, 20% as ‘good’, and 2% as ‘pleasurable’. Assessment of professionals’ evaluation revealed a 60%-acceptability according to ‘very good’ and 40% ‘good’.

During the intervention period, three participants (one of RT and two of CT) abandoned participation due to reasons of relocation to another care home.

## 4. Discussion

This study compared RT with more commonly used CT in care home residents with MCI. We demonstrated that RT is not inferior to CT and provide initial evidence of possible superiority for positively affecting cognitive and emotional status when a combination of RT and CT is offered. In this study we aimed to answer research questions considering the evaluation and development of effective non-pharmacological treatment in gerontology. The main body of literature in MCI-treatment focuses on cognitive outcome criteria regardless of emotional outcome parameters that are likely to influence cognitive criteria [19]. Until now, little has been known about the broader effects aside from cognitive performance produced by RT compared to CT. To the best of our knowledge, this was the first study to compare RT and CT and further appended a combined training program in older adults with MCI.

### 4.1. Cognitive Status

Both groups achieved a clinically relevant decline in cognitive deficit severity, shifting from mild cognitive deficits (MMSE score 26–21 points) to a score being considered as normal (MMSE score 30–27 points). Regarding concentration and vigilance, participants of both groups have been located in normal range at baseline. However, only RT, and not CT, led to additional benefits in the cognitive status in two of totally eight sub-areas analyzed: in the sub-area ‘attention and calculation’ (MMSE) and in ‘work pace’ (ACT), RT was superior to CT over the total training period. It has been shown previously that group interventions for AD patients without time or performance pressure positively influences cognitive performance [37]. Our findings are in line with previous research results, since training interventions combining cognitive elements with relaxation training have shown to lay the foundation for raising memory performance [38]. In cognitive training interventions only, stress is likely to occur [37]. Therefore, elements promoting relaxation are particularly important in cognitive training interventions for older adults due to stress minimizing effects. 

### 4.2. Emotional Status

As expected, the training interventions resulted in positive alterations in self-reported well-being/mood disturbance (CSRS-AMS) and fewer depressive symptoms (GDS-SF). After the first training period of RT and CT, as well as after the combined training period, both groups experienced significant improvements in emotional status. Nevertheless, RT did not bring any additional benefits compared to CT as hypothesized. This result could reflect a floor effect, since participants were not in a clinically relevant domain of depression at baseline. The findings for enhanced emotional status are consistent with previous results reporting increasing well-being through interventions rich in variety, joy and positive interaction and relaxed atmosphere [12,39,40]. Furthermore, the application of music has previously shown to result in positive impact on cognition and affective aspects in particular and could therefore be considered for future research [26,41].

### 4.3. Experience of Pain

The overall training period resulted in a significant decrease in the experience of pain, that is considered especially relevant since 58% (RT) and 67% (CT) of participants took pain medication on a regular basis. The acute application of pain medication by participants, which might be of particular interest for the clinical needs, could not be monitored in order to verify results due to objective parameters. 

### 4.4. Limitations

Due to organizational issues in the field of gerontological treatment, some limitations have to be addressed. Firstly, it was not possible to establish a wash-out phase between the first and second training period (between t2 and t3). Therefore, it might be possible that carry-over-effects from the training program in the first period accidentally appeared as resulting effects of the combined training program in the second training period. Secondly, no passive control group was implemented due to ethical reflections on withholding potentially effective treatment, especially considering previous research results on passive control groups. Thirdly, a classical cross-over study design was inapplicable due to our hypothesis of further benefits attributable to the combined training program. Fourthly, the duration of the two intervention periods, respectively, three weeks each, was set rather short. In the fifth place, authors were aware of likely influence of trainer- and group leader effects such as the skewing Pygmalion effect. 

## 5. Conclusions

Cognitive training and relaxation training have shown to be appropriate to enhance cognitive and emotional health. We further observed that a subsequent three-week combined training program additionally enhanced cognitive and emotional status and decreased experience of pain. In general, relaxing and stress reducing elements should be regarded as more effective and successful treatment methods for prevention and rehabilitation in gerontology and dignified aging than has formerly been the case. 

## Figures and Tables

**Table 1:** Comparison of participant’s sociodemographic and clinical data in both groups.

Variable	RT	CT
Participants (%)	26 (52%)	24 (48%)
Age: M ± SD	87 ± 7	87 ± 5
Number of female/male	25/1	24/0
**Education level**	**%**	**%**
Primary school	38	25
Apprenticeship	35	17
University-entry diploma	19	41
University degree	8	17
**Family Status**	**%**	**%**
Single	31	21
Married or in a Partnership	8	17
Widowed	61	62
**Level of care**	**%**	**%**
Level 1–4 (no permanent care needed)	65	75
Level 5–7 (increased & permanent care needed)	35	25
**Medication Status**	**%**	**%**
psychotropic medication	11	8
medication for physical illness	58	67
combined psychotropic- & medication for physical illness	23	17
no medication	8	8

M = mean, SD = standard deviation, RT = Relaxation training, CT = Cognitive training.

**Table 2:** Changes in cognitive status (MMSE, ACT) and emotional status (mood disturbance/well-being (CSRS-AMS) and depressive symptoms (GDS-SF score)).

	Descriptives M (SD)						Inferential					
	t1		t2		t3			*p*-Value			Partial η^2^	
Variables	M	SD	M	SD	M	SD	Group	Time	Inter-Action	Group	Time	Inter-Action
**Cognitive Status**												
*Mini-Mental State Examination (MMSE)*												
*MMSE total score*							0.261	**<0.001**	0.099	0.03	**0.28**	0.05
RT	24.8	(3.5)	26.4	(3.0)	26.8	(2.2)						
CT	26.3	(2.7)	26.9	(2.8)	27.4	(2.3)						
*MMSE orientation*							0.252	**0.008**	0.786	0.03	**0.10**	0.01
RT	9.1	(1.2)	9.4	(0.8)	9.7	(0.6)						
CT	8.9	(1.0)	9.3	(1.1)	9.3	(0.8)						
*MMSE registration*							0.701	0.781	0.349	<0.01	<0.01	0.02
RT	3.0	(0.5)	3.0	(0.2)	3.0	(0.0)						
CT	3.0	(0.2)	3.0	(0.0)	3.0	(0.0)						
*MMSE attention and calculation*							**0.045**	**0.006**	**0.038**	**0.08**	**0.10**	**0.07**
RT	3.6	(1.6)	4.1	(1.0)	4.4	(0.8)						
CT	4.5	(0.8)	4.5	(1.1)	4.7	(0.7)						
*MMSE recall*							0.359	**0.002**	0.238	0.02	**0.12**	0.03
RT	1.5	(1.2)	2.1	(1.7)	2.0	(0.9)						
CT	1.8	(1.0)	2.0	(0.9)	2.4	(0.7)						
*MMSE language*							0.262	0.345	0.695	0.03	0.02	0.01
RT	7.5	(1.0)	7.8	(1.3)	7.9	(0.8)						
CT	7.9	(1.1)	7.9	(1.0)	8.0	(1.0)						
*Age-Concentration-Test (ACT)*												
*ACT overall score*							0.143	0.108	0.717	0.04	0.05	<0.01
RT	3.8	(0.4)	3.9	(0.1)	3.9	(0.1)						
CT	3.9	(0.2)	4.0	(0.1)	4.0	(0.9)						
*ACT work pace*							0.370	**<0.001**	**0.026**	0.02	**0.19**	**0.08**
RT	4.6	(0.8)	4.5	(0.7)	4.4	(0.7)						
CT	4.5	(0.6)	4.5	(0.6)	4.2	(0.6)						
*ACT correct answer*							0.662	0.153	0.211	<0.01	0.04	0.03
RT	2.8	(0.2)	2.9	(0.1)	2.9	(0.2)						
CT	2.8	(0.3)	2.9	(0.2)	2.9	(0.2)						
*ACT percentage of mistakes*							0.155	**0.024**	0.765	0.04	**0.08**	0.01
RT	2.6	(1.0)	2.8	(0.7)	2.8	(0.6)						
CT	2.6	(0.8)	2.1	(0.5)	2.3	(0.9)						
**Emotional Status**												
*Clinical Self-Rating Scale (CSRS-AMS)*							0.613	**<0.001**	0.376	0.01	**0.30**	0.02
RT	14.7	(9.7)	11.0	(7.8)	8.7	(6.9)						
CT	12.5	(7.6)	10.9	(5.5)	8.2	(5.4)						
*Geriatric Depression Scale—Short Form (GDS-SF)*							0.801	**<0.001**	0.325	<0.01	**0.28**	0.02
RT	3.1	(3.0)	1.9	(2.4)	1.4	(1.5)						
CT	2.8	(2.1)	2.3	(1.6)	1.6	(1.6)						
**Experience of Pain**												
*Visual Analog Scale (VAS) Measurement of Pain*							0.542	**0.019**	0.681	0.01	**0.08**	0.01
RT	2.8	(3.1)	2.9	(3.2)	2.2	(2.6)						
CT	3.6	(3.0)	3.2	(2.6)	2.3	(2.2)						

RT = first relaxation training, then combined training (n = 26); CT = first cognitive training, then combined training (n = 24), M = mean, SD = standard deviation; bolded numbers indicate statistically significant values.

## Data Availability

The data in this study may be requested from the corresponding author.
